# The Horse’s Foot1Read at a meeting of the Veterinary Medical Association of New Jersey, held at Trenton, January 9, 1902.

**Published:** 1902-03

**Authors:** James T. McDonough

**Affiliations:** Montclair, N. J.


### THE HORSE'S FOOT.[1]

By James T. McDonough, V.S.,
MONTCLAIR, N. J.

After first thanking you for this privilege and apologizing for my inability to properly treat so important a subject, I will state that the object of my paper is to create a discussion on that long-

[1] Read at a meeting of the Veterinary Medical Association of New Jersey, held at Trenton, January 9, 1902.

neglected and very important organ—the horse's foot and its shoe of nature, the hoof.

I know that for several reasons this has always been a very complex subject for veterinarians to deal with. First of all, owners are not conscious of the fact that most lameness and injuries of the limb can be traced to the foot; second, they do not know that a horse is lame unless he is lamer in one leg than in the other—in other words, unless a horse travels with a perceptible limp our most humane people will compel him to go, unconscious of his sufferings; third, it has long been the custom for the care of the feet to be left to the farrier, and as to just where his duties end and those of the veterinarian begin has always been a problem that neither could solve.

As to the first cause, we are responsible for its existence. If we are called to see a lame horse and find him suffering with a sprained tendon or ligament, a soreness of some muscle or muscles, bony enlargement, etc., we do just what any owner or groom would do —simply treat the part affected, which in most cases they have found and suggested the same treatment that we will apply, and oftentimes will themselves apply it with just as satisfactory results. Now we as veterinarians know that the cause of the lameness was in itself an effect, and it is our duty to find and remove its cause, for by so doing we not only prevent a recurrence of the trouble, but we satisfy the owner that our knowledge of those things is superior to his, as the condition can oftentimes be relieved, and permanently so, without the application of any treatment to the parts affected than by simply removing the cause—a service that can only be performed by the veterinarian, who has a knowledge of the anatomy of the foot and limb and the physiology of motion.

The second cause can easily be overcome after having successfully removed the first, for in most cases the owners, after once being convinced that a condition could exist unnoticed that would result in an injury to the limb and lameness, will seek our opinion of their other horses, when we can advise them of their condition, and by our skill relieve them, and allow them to travel with a freedom that will be noticed and appreciated by the owner.

The third cause: It is the duty of the farrier to perform the work of horseshoeing. There is no one who can take his place, and it is far from my object to make little of his service. He is held responsible for all kinds of lameness, and while I do not wish to infer that he is entirely blameless, I do say, with authority, that there would be more lame horses if the judgment he exercises was commensurate to the pay he receives. As to where his duties end and those of the veterinarian begin, why they end just where his knowledge of the subject ends, and it is right there where the veterinarian's begins. Their duties are different and distinct. The one must not become a consulting shoer, and the other cannot be considered a consulting veterinarian. But, to get back to the subject of my paper and make clear its object, we will consider the relation of the hoof to the foot and their relations to the rest of the limb.

The shell, as we know, surrounds and protects the foot just as our shoes surround and protect our feet. It is the shoe of nature in the full sense of the term, and it is fitted so nicely and accurately to the foot as to permit of no alteration in its shape without causing pain and discomfort to that most sensitive organ. If this statement be true, we have only to notice the distorted shape of nearly all hoofs to form some opinion of the suffering endured by those animals that are powerless to relieve it. They are not even allowed to rest that they might lessen it. They manifest unmistakable symptoms of pain. They go sore, point first one foot, then the other. They move along, oftentimes under the whip, with little, short steps; but it is only when the pain of one foot is more intense than that of the other, which causes them to limp, that we respond to their pleadings for relief, and then only to the extent of restoring the crippled limb to its former usefulness.

But our duty does not end there. We should know that the shape of that hoof cannot insure comfort to the foot, and it becomes our duty either to shape it or to direct the performance of that work. The relation of the foot to the limb, and the different parts of the limb to each other, depends entirely upon the shape of the shell. This I wish to make plain.

We know that the possibility of a crooked and flexible column of bones to support the weight of a horse's body depends upon the relation of one to the other and the support they receive from the muscles, tendons, and ligaments that enter into the formation of the limb.

We also know that if we place a column of bones in a vertical position, with the first firmly attached to a base, that the relation of the ends of those bones to each other will depend upon the shape of the base and its position upon the ground, and, as the hoof forms the base of this complete column of sensitive tissue, we can readily see that any alteration of its normal shape can only result in an injury to the limb. But man has altered all of them. You probably have few normal-shaped hoofs in this city to-day, and the only reason that more horses are not lame is because the work they perform does not tax the distorted limb to the limit of its endurance. But while it is only reasonable to suppose that the limbs were intended to perform work proportionate to the other organs—heart, lungs, etc.—yet we know if subjected to violent exercise, such as trotting or running, the legs or feet are the first to give out. In fact, it is generally believed by owners of fast horses that their limbs are unequal to the task of performing this work; and yet I do not know of a single injury to the limb below the knee, unless from some external cause, that the cause cannot be traced to the hoof, and must be removed before the injury can be permanently relieved.

If we are called to see a horse suffering with some injury to a muscle, tendon, or ligament, not traceable to any external cause, we must account for it in one of two ways—that it was either unequal to the task of performing its work, or there was imposed upon it more work than it was intended to perform. As to which it was we can be reasonably sure by examining the same part of the opposite limb and finding it free from injury. But if the work of this part of the limb has been increased, that of some other part or parts must have been lessened to the same extent, and as this transfer of work from one part of the limb to another can only be caused by a change in the relation of the parts to each other, and as we know the relation of those parts depends upon the shape and position of its base—the hoof—it would seem that we would have to seek relief for our trouble at that point.

Again, it is an indisputable fact that owners of some runners have been known to wait for their quarters to break before expecting them to win a race; and there is no question but some of those horses have run faster with broken and bleeding quarters than they could with the hoof intact. Now, anyone knows that the breaking of that hoof and the rupturing of that most sensitive tissue—the laminæ—cause pain to the animal; but the pain it caused was less than that caused by the distorted shape of the hoof, which was responsible for the quarter crack. But, gentlemen, I know that I have taxed your patience to the limit of its endurance, and will conclude by thanking you for your kind attention and requesting a liberal exchange of views upon the subject of my paper.

